# Effects of Ketoconazole on ACTH-Producing and Non-ACTH-Producing Neuroendocrine Tumor Cells

**DOI:** 10.1007/s12672-019-00361-6

**Published:** 2019-05-18

**Authors:** Aura D. Herrera-Martínez, Richard A. Feelders, Wouter W. de Herder, Justo P. Castaño, María Ángeles Gálvez Moreno, Fadime Dogan, Rosanna van Dungen, Peter van Koetsveld, Leo J. Hofland

**Affiliations:** 1 Department of Internal Medicine, Division of Endocrinology, Erasmus MC, Wytemaweg 80, 3015 CN Rotterdam, The Netherlands; 2 Maimonides Institute for Biomedical Research of Cordoba (IMIBIC), Córdoba, Spain

**Keywords:** Ketoconazole, Combined therapy, Neuroendocrine tumors, Proliferation, Secretion

## Abstract

Prolonged remission of hypercortisolism with steroidogenesis inhibitors has been described in patients with ectopic adrenocorticotropic hormone (ACTH) syndrome. The anti-proliferative and pro-apoptotic effect of ketoconazole in human cancer cells was previously suggested. The aim of this study was to explore the effects of ketoconazole on ACTH-producing and non-ACTH-producing neuroendocrine tumor (NET) cell lines. The effects of ketoconazole alone, and in combination with somatostatin analogs, were evaluated in two human cell lines: DMS-79 (ectopic ACTH-producing small cell lung carcinoma) and BON-1 (human pancreatic NET). Total DNA measurement, apoptosis, cell cycle, chromogranin A (CgA)/proopiomelanocortin (POMC) expression by qRT-PCR, serotonin, CgA, and ACTH secretion assays were performed. In both cell lines, ketoconazole significantly suppressed cell growth and colony formation in a dose and time-dependent manner. The effect in DMS-79 was primarily cytotoxic, while it was more apoptotic in BON-1 cells. Ketoconazole also induced increase in G0/G1 phase in both cell lines and arrest in phase G2/M of BON-1 cells. Ketoconazole did not affect the secretion of serotonin, CgA, ACTH, or the mRNA expression of CgA and POMC. Decreased serotonin secretion was observed after the combination treatment with pasireotide. These results suggest a direct effect of ketoconazole on cell proliferation, apoptosis, and cell cycle in both ACTH- and non-ACTH-producing NET cells.

**Electronic supplementary material:**

The online version of this article (10.1007/s12672-019-00361-6) contains supplementary material, which is available to authorized users.

## Background

Neuroendocrine tumors (NETs) are a heterogeneous family of neoplasms derived from neuroendocrine cells with a wide spectrum of clinical behavior [1]. NETs can occur sporadically or as a result of hereditary predisposition syndromes [2]. Their capacity to secrete peptide hormones divides them in functioning and non-functioning tumors [1].

One of the most uncommonly secreted hormones is adrenocorticotropic hormone (ACTH) [3, 4], resulting in hypercortisolism and ectopic ACTH syndrome (EAS). EAS is a form of Cushing syndrome (CS) associated with overt malignancies or indolent tumors, including neuroendocrine tumors of the lungs, thymus, and gastrointestinal tract [3–6]. The management of patients with EAS requires strict control of hypercortisolism as soon as the diagnosis is established, in order to avoid related complications [3, 7, 8]. In EAS patients, initial resection of the primary lesion is the first-line treatment option. However, in some cases, surgery is not possible or successful and in other cases, the source of ectopic ACTH is not identified [8, 9]. For these patients, medical treatment with steroidogenesis inhibitors including ketoconazole and bilateral adrenalectomy represents alternative therapeutic options [10–12].

Ketoconazole is widely used for medical treatment of CS and can improve clinical signs, symptoms, and comorbidities [10]. Ketoconazole impairs adrenal and gonadal steroidogenesis by inhibiting side-chain cleavage, 17,20-lyase, and 11-β hydroxylase enzymes [13]. Because cortisol production is often very high in EAS, ketoconazole may be less effective in controlling hypercortisolism in EAS patients compared with Cushing disease patients [10]. However, the drug could exert additive effects in the control of patients with severe hypercortisolemia [13]. A direct effect of ketoconazole on tumoral ACTH secretion has been suggested [14, 15], since its use in some EAS cases has been followed by prolonged remission of hypercortisolemia [14–16]. Ketoconazole also reduces ACTH secretion from thymic tumor cells in culture [17], suggesting a dual (direct/indirect) action of ketoconazole on EAS tumors.

Some ectopic ACTH-producing tumors express somatostatin receptors, suggesting a putative role of somatostatin analogs (SSA) for reducing ACTH production. Octreotide inhibits the release of ACTH in some patients with Nelson’s syndrome and EAS from metastatic gastroenteropancreatic-NETs, small cell lung carcinoma, carcinoids, and medullary thyroid carcinoma [18–20]. However, in clinical practice, the effect of SSA is limited, but their use has been suggested when other inhibitors of steroidogenesis fail or when parenteral administration is required [21].

Despite the development of new therapeutic options in NETs, treatment strategies are still limited. As such, it is necessary to develop novel therapeutic strategies. In this scenario, we aimed to evaluate additional, not previously described in vitro effects of ketoconazole as monotherapy, as well as in combination with SSA on proliferation and hormonal secretion of ACTH-producing and non-ACTH-producing neuroendocrine tumor cell lines, in order to determine potential off target effects of ketoconazole on NET cells.

## Materials and Methods

### Cell Culture

We used the human small cell lung carcinoma cell line DMS-79 and the human pancreatic neuroendocrine tumor cell line BON-1. The cell line DMS-79 (ATCC-CRL-2049™, Manassas, VA, USA) was isolated from pleural fluid of an ACTH-producing small cell lung carcinoma. The BON-1 cell line was a kind gift of Dr. Townsend (The University of Texas Medical Branch, Galveston, TX, USA) and was established from a lymph node metastasis of a human functional pancreatic neuroendocrine tumor. Both cell lines were routinely cultured in 75- cm^2^ flasks (Greiner bio-one, The Netherlands) at 37  °C in a 5% CO_2_ incubator.

BON-1 cells were cultured in DMEM/F12 (GIBCO Biocult Europe, Breda, The Netherlands) containing 10% FCS, l-glutamine, fungizone (0.5 mg/L), and penicillin (100 U/mL) (Bristol-Myers Squibb, Woerden, The Netherlands). Cells were harvested with trypsin (0.05%)–EDTA (0.53 mM) and resuspended in culture medium. Cell viability always exceeded 85%. For the serotonin and chromogranin measurements, BON-1 cells were cultured in DMEM/F12 (GIBCO Biocult Europe, Breda, The Netherlands) containing 0.1% BSA, l-glutamine, fungizone (0.5 mg/L), and penicillin (10^5^ U/L) (Bristol-Myers Squibb, Woerden, The Netherlands) after an initial incubation period of 24 h in 10% FCS to allow the cells to attach.

DMS-79 cells were cultured in RPMI 1640 (GIBCO Biocult Europe, Breda, The Netherlands) containing 10% heat-inactivated (Hi)-FCS, l-glutamine, and penicillin (10^5^ U/L) (Bristol-Myers Squibb, Woerden, The Netherlands). Cells were harvested with pipetting and cell viability always exceeded 80%. All cell line proliferation experiments were performed at least four times.

### Drugs and Reagents

Ketoconazole was purchased from Sigma-Aldrich (Zwijndrecht, The Netherlands) and dissolved in ethanol as a 10^−2^ M stock solution (stored at − 20 °C) and diluted in ethanol prior to use. We tested concentrations ranging between 10^−5^ and 10^−7^ M in BON-1 cells and concentrations ranging between 5 × 10^−5^ and 5 × 10^−7^M in DMS-79 cells. Control wells were treated only with vehicle only (ethanol). We used different concentrations for both cell lines because the cell lines showed a different sensitivity to ketoconazole in pilot experiments (not shown). For both cell lines, all experiments included the clinically relevant concentrations of ketoconazole.

Octreotide and pasireotide were obtained from Novartis Pharma (Basel, Switzerland) and diluted in medium until a final concentration of 10^−8^ M.

### Cell Proliferation Assay

#### Measurement of Total DNA Content (Measure of Total Cell Number)

Cells were plated in 1 mL medium in 24-well plates at a density necessary to obtain a 65–70% cell confluence in the control groups at the end of the experiment (50,000 cells/well for 3 days and 10,000 cells/well for 7 days in BON-1 cells; 150,000 cells/well for 3 days and 75,000 cells/well for 7 days in DMS-79 cells). Twenty-four hours later, the medium was refreshed and increasing concentrations of ketoconazole, alone or combined with SSA, were added to the cells.

After 3 and 7 days of treatment, the cells were harvested for DNA measurement, as a measure of cell number. For 7 days, treatment medium and drugs were refreshed at day 3. The procedure for the DNA measurement has been previously described in detail [22, 23]. Briefly, the cells were treated with 150 μL of ammonia solution (1 mol/L)-Triton X 100 (0.2% *v*/*v*) for 15 min. Thereafter, sonification was performed (Soniprep 150; amplitude 1400 μm). Subsequently, 1 mL of assay buffer (100 mM NaCl, 100 mM EDTA, 10 mM Tris; pH 7.0) was added. For DNA measurement, 20 μL of the solution was mixed with 200 μL of Hoechst dye H33258 solution (1 μg/mL). Fluorescence was measured with the excitation and emission wavelengths set at 350 and 455 nM respectively. The fluorescence of experimental samples was referenced to a standard curve of calf thymus DNA (type II, no D-3636; Sigma-Aldrich (Zwijndrecht, The Netherlands).

#### Soft Agar Colony Forming Assay

The effect of ketoconazole on colony formation was evaluated using a soft agar colony forming assay. The method was according to a previously described protocol [24]. The number of cells plated in the upper layer of 0.3% agar (Sigma Aldrich) was 15,000 for DMS-79 and 5000 for BON-1. After 7 and 14 days, 1 mL of medium without or with increasing concentrations of ketoconazole (range between 5 × 10^−5^ and 10^−6^ M) was added. Adequate colony formation was achieved around day 21. At this time point, the cells were stained with 200 μL nitroblue tetrazolium chloride solution (1 mg/mL; Sigma) per well and overnight incubation at 37 °C. Once colonies were stained, photomicrographs were taken with a Multimage Light Cabinet (Alpha Innotech Corporation, USA). Analysis of colony number and size was performed using the software program ImageJ. Only colonies with a size ≥ 75 μM were analyzed.

#### Quantitative RT-PCR

mRNA expression of chromoganin A (CgA) in BON-1 cells, proopiomelanocortin (POMC) in DMS-79 cells, and somatostatin receptors (SST_1_, SST_2_, SST_3_, SST_5_) in both cell lines was evaluated by quantitative RT-PCR. We used a previously described method [25]. In short, poly(A^+^) mRNA was isolated using Dynabeads Oligo (Deoxythymidine)_25_ (Dynal AS, Oslo, Norway). The poly(A^+^) mRNA was eluted in H_2_O (65 °C) twice for 2 min each and used for cDNA synthesis in a Tris buffer [50 mm Tris-HCl (pH 8.3), 100 mm KCl, 4 mm dithiothreitol, and 10 mm MgCl_2_] with 10 U ribonuclease inhibitor, 2 U avian myeloblastosis virus Super Reverse Transcriptase, and 1 mm of each deoxynucleotide triphosphate in a final volume of 40 μL. This mixture was incubated for 1 h at 42 °C, and the resulting cDNA was diluted fivefold in 160 μL sterile H_2_0. The total reaction volume (25 μL) consisted of 10 μL cDNA and 15 μL TaqMan Universal PCR Mastermix (Applied Biosystems, Branchburg, NJ). Primers were used at final concentration of 300 nm and probe at 200 nm. Real-time qPCR was performed in 96-well optical plates with the TaqMan Gold nuclease assay (Applied Biosystems, Roche) and the ABI Prism 7700 Sequence Detection System (PerkinElmer, Foster City, CA). After two initial heating steps at 50 °C (2 min) and 95 °C (10 min), samples were subjected to 40 cycles of denaturation at 95 °C (15 s) and annealing at 60 °C (60 s). All samples were assayed in duplicate. Dilution curves were constructed to calculate PCR efficiencies (E) for every primer-probe set [26]. To exclude genomic DNA contamination in the RNA, the cDNA reactions were also performed without reverse transcriptase and amplified with each primer pair. To exclude contamination of the PCR mixtures, the reactions were also performed in the absence of a cDNA template.

CgA and POMC mRNA were normalized to the housekeeping genes *HPRT1*, beta-glucuronidase (*GUSB*), and beta-actine (*ACTB/ACTB*) expression levels using the method of Vandesompele [27]; somatostatin receptors were normalized against *HPRT1*. *CgA*, *POMC*, *GUSB*, *ACTB* primer/probes were purchased from Thermo Fisher Scientific, The Netherlands, while the used sequence of somatostatin receptors and *HPRT1* has been previously described [28]. PCR efficiencies were as follows: *CgA*: 1.95, *POMC*: 1.92, *SST*_*1*_: 2, *SST*_*2*_: 1.91, *SST*_*3*_: 1.92, *SST*_*5*_: 1.92, *HPRT1*: 1.91, *ACTB*: 1.91, *GUSB*: 2. The relative expression of genes was calculated using the comparative threshold method, 2^−ΔC1^ [29], after efficiency correction [30] of target and reference gene transcripts (*HPRT*, *GUS-B*, *ACTB*). All experiments were performed at least twice with four replicates.

#### Cell Cycle Assay—Apoptosis Analysis

Cells were plated in 12-well plates at the density necessary to obtain a 65–70% cell confluence in the control groups at the end of the experiment. Twenty-four hours later, ketoconazole was added to wells in triplicate. In each cell line, the effects of ketoconazole on cell cycle were tested in three concentrations (5 × 10^−6^, 10^−6^, and 5 × 10^−5^ M in DMS-79; 10^−6^, 5 × 10^−6^, and 10^−5^ M in BON-1 cells) on cell proliferation after 7 days of treatment. We evaluated the effects of the compounds on cell cycle and apoptosis after 72 h of treatment. Following treatment, cells were harvested and collected by centrifugation. For cell cycle, cells were fixed in 70% ice-cold ethanol, followed by an overnight incubation at − 20 °C. Samples were measured using the Muse™ cell cycle kit (Merck) and prepared according to the manufacturer’s protocol. For apoptosis measurement, cells were resuspended in 100 μL PBS/1% FCS, according to the protocol provided by the manufacturer. A commercially available kit was used (Muse™ annexin V dead cell). All measurements were performed using the Muse® Cell Analyzer (Merck). Apoptosis was measured using a commercially available ELISA (Cell Death Detection ELISA Plus, Roche, The Netherlands) according to the manufacturer’s protocol. Lactate dehydrogenase LDH release was measured using a kit (pierce LDH cytotoxicity assay kit, Thermo scientific, The Netherlands) and samples were treated according to the protocol provided by the manufacturer. All experiments were performed at least three times using three replicates.

#### ACTH Secretion Assay

In DMS-79 cells, we evaluated the effects of ketoconazole alone and in combination with octreotide/pasireotide on ACTH secretion after 3 and 7 days of treatment. Cells were plated in 24-well plates and treated as described earlier for the cell proliferation assay. The experiment was performed twice. ACTH levels were determined using a chemiluminescent immunometric assay in an IMMULITE® 2000 system. ACTH secretion values were normalized for cell amount per well (DNA). The sensitivity of this assay ranged between 1.1 and 270 pmol/L. Untreated control values were within this range. All experiments were performed at least two times using four replicates.

#### Chromogranin A and Serotonin Secretion Assay

BON-1 cells were plated in 24-well plates using a medium containing 0.1% BSA, and treated as described earlier for the cell proliferation assay. Medium was collected from each well, to samples for the serotonin ELISA ascorbic acid was added (final concentration 0.1%). A commercially available human chromogranin An ELISA kit (Epitope diagnostics Inc., San Diego CA, USA) and a serotonin high sensitive ELISA (IBL International, Hamburg Germany) were used following the manufacturer’s instructions. The DNA content per well was used to correct both serotonin and CgA absolute values. All experiments were performed at least two times using four replicates.

#### Statistical Analysis

For the statistical analysis, statistical software of GraphPad Prism version 5 (GraphPhad Software, San Diego, CA) was used. Between-group comparisons were analyzed by the Mann–Whitney *U* test (nonparametric data), or the Kruskal–Wallis test (nonparametric data, when we compared more than two groups). Differences were taken to be statistically significant at *p* < 0.05. Results are expressed as mean ± S.E.M. and percentages. Log transformation was used for calculating the IC_50_.

## Results

### Effects of Ketoconazole

**Fig. 1:**
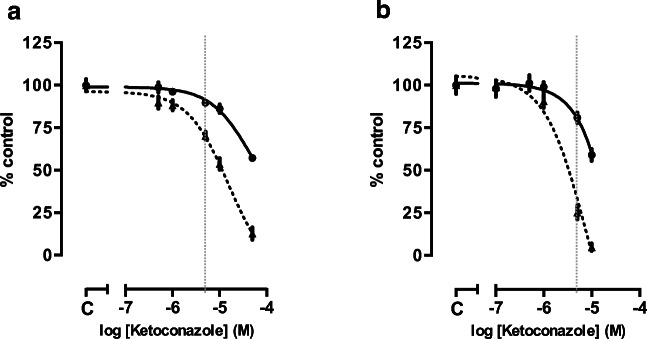
Dose- and time-dependent effect of ketoconazole after 3 and 7 days of treatment. **a** Effect on cell growth (DNA) in DMS-79 cells. **b** Effect on cell growth in BON-1 cells. Ketoconazole significantly suppressed cell growth in a dose- and time-dependent manner. Solid and dotted black lines represent DNA content in percentage after 3 (__) and 7 days (…) respectively. The gray vertical dotted line represents the clinically relevant plasmatic concentration of ketoconazole. Values represent mean ± SEM and are shown as a percentage of control. **c** Control

The growth inhibitory effects of ketoconazole on DMS-79 and BON-1 cells growth are dose- and time-dependent (Fig. 1).

**Fig. 2:**
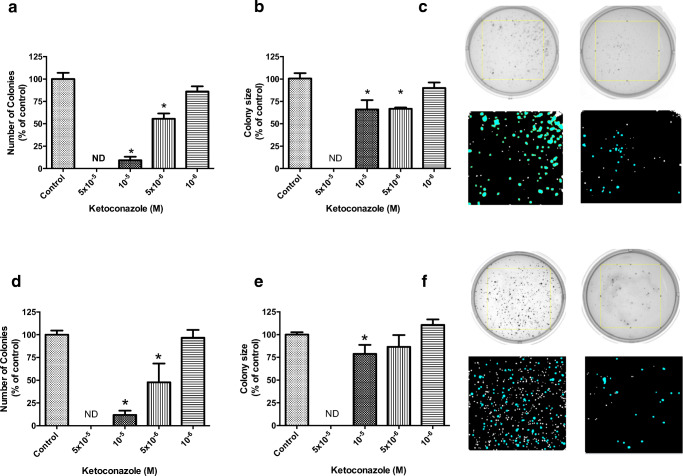
Dose-dependent effect of ketoconazole on colony formation. **a** Colony number, **b** colony size of DMS-79 cells. **d** Colony number, **e** colony size of BON-1 cells. Ketoconazole significantly suppressed the number and size of colonies in both cell lines. Right panels (**c**, **f**) show representative images of the colonies treated with 5 × 10^−6^ M ketoconazole after staining with nitroblue tetrazolium chloride and analysis of images with ImageJ. Values represent mean ± SEM and are shown as a percentage of vehicle-treated control. Asterisks *, *p* < 0.05. ND non-detectable

DMS-79 cells (Fig. 1a) are slightly less sensitive to ketoconazole compared with BON-1 cells (Fig. 1b). The effects of ketoconazole ranged between 47.20% inhibition (*p* < 0.0001) at the maximal dose (5 × 10^−5^ M) and 10.32% (*p* < 0.05) at the dose of 5 × 10^−6^ M after 3 days of treatment. After 7 days, the effect of ketoconazole increased to 87.3%, 46.3%, and 29.8% with ketoconazole 5 × 10^−5^ M, 10^−5^ M, and 5 × 10^−6^ M respectively (*p* < 0.0001). The IC50 after 7 days of incubation was 1.5 × 10^−5^ M (95%CI 1.0 × 10^−5^–2.3 × 10^−5^ M). Ketoconazole (10^−5^ M and 5 × 10^−6^ M) significantly inhibited the number (90.73–44.41%, respectively) and the size (33.95–33.30%, respectively) of the colonies in agar (Fig. 2a). 5 × 10^−5^ M ketoconazole completely abolished DMS-79 colony formation.

In BON-1 cells, the inhibitory effect of ketoconazole ranged between 41.03% at the maximal dose (10^−5^ M; *p* < 0.0001) and 19.1% at a dose of 5 × 10^−6^ M (*p* < 0.01) after 3 days of treatment (Fig. 1b). After 7 days, the growth inhibitory effect increased to 95.23% and 74.82% with ketoconazole 10^−5^ M and 5 × 10^−6^ M respectively (*p* < 0.0001) (Fig. 1b). The IC50 after 7 days of incubation was 7.7 × 10^−6^ M (95%CI 3.8 × 10^−6^–1.6 × 10^−5^ M). Ketoconazole (10^−5^ M and 5 × 10^−6^ M) significantly inhibited the number (88.31–52.19%, respectively). Only ketoconazole 10^−5^ M decreased the colony size of this cell line in agar (21.2%) (Fig. 2b). Ketoconazole 5 × 10^−5^ M completely abolished BON-1 colony formation.

### Effect of Ketoconazole in Combination with Octreotide and Pasireotide

**Fig. 3:**
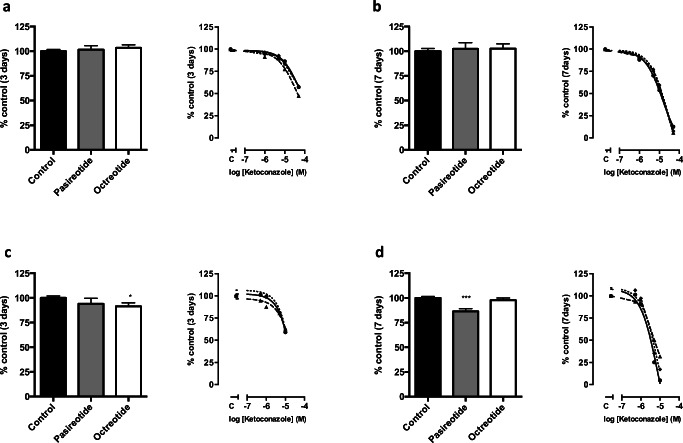
Dose- and time-dependent effects of the combination treatment with ketoconazole and somatostatin analogs on cell proliferation after 3 and 7 days of treatment. **a**, **b** Percentage of control of cell growth in DMS-79 cells after 3 and 7 days respectively; **c**, **d** percentage of control of cell growth in BON-1 cells after 3 and 7 days respectively. The bar graphs represent the effect of SSA alone (octreotide and pasireotide, both at 10^−8^ M), compared with untreated control. The line graphs represent the effect of ketoconazole alone, or in combination with a fixed dose (10^−8^ M) of SSA (set at 100%). Ketoconazole significantly suppressed DNA content in a dose and time-dependent manner, a slightly decreased effect was observed after a combination with somatostatin analogs. Lines represent DNA content as a percentage after incubation with ketoconazole alone (solid line), ketoconazole-octreotide (dashed line), and ketoconazole-pasireotide (dotted line) respectively. Values represent mean ± SEM and are shown as a percentage of control. C, control. Asterisks *p < 0.05; ***p < 0.0001 compared with untreated controls

In DMS-79 cells, neither pasireotide nor octreotide inhibited cell growth (Fig. 3a and b, left panels). In combination with pasireotide or octreotide, ketoconazole exerted a similar growth inhibitory effect compared with the effect of ketoconazole alone (Fig. 3a and b, right panels).

In BON-1 cells, octreotide slightly decreased cell growth by 9.4% (*p* < 0.05) after 3 days of treatment only (Fig. 3c, left panel), whereas pasireotide slightly inhibited cell growth by 13.5% (*p* < 0.01) at day 7 (Fig. 3d, left panel). In combination with pasireotide and octreotide, ketoconazole appeared slightly less effective at high dose ketoconazole (Fig. 3c and d, right panels).

### Somatostatin Receptor Subtype Expression

The relative mRNA expression of somatostatin receptor subtypes was evaluated in both cell lines (Supplementary Figure 1). SST_1_ and SST_5_ expression in DMS-79 was lower compared with BON-1 cells. Rank order of expression in DMS-79 was SST_1_ = SST_2_ > SST_3_ > SST_5_ and for BON-1 SST_1_ > SST_5_> > SST_2_ > SST_3_.

### Apoptosis Assays

**Fig. 4:**
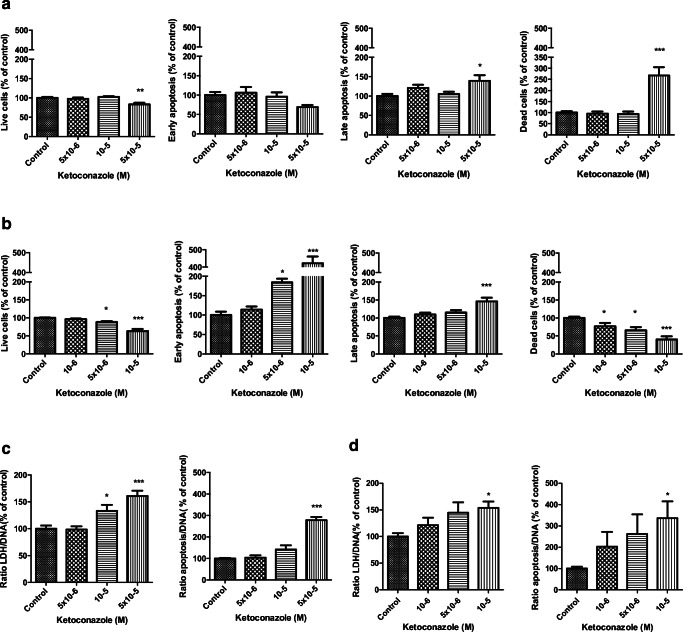
Dose-dependent effect of ketoconazole on apoptosis after 3 days of treatment. **a** DMS-79 cells: the highest ketoconazole concentration tested (5 × 10^−5^ M) induced a significant increase in dead cells and late apoptosis. **b** BON-1 cells: the highest ketoconazole concentration tested (10^−5^ M) induced a significant increase in early apoptosis as well as in late apoptosis. Apoptosis (DNA fragmentation) and LDH release after 3 days of treatment in DMS-79 cells (**c**) and BON-1 cells (**d**). The highest ketoconazole concentration tested increased apoptosis as well as LDH levels in both cell lines. Values represent mean ± SEM and are expressed as percentage of control of the apoptosis/DNA ratio and LDH/DNA ratio. Asterisks *p < 0.05; **p < 0.01, ***p < 0.001 compared with untreated controls

In DMS-79 cell line, the highest ketoconazole concentration evaluated (5 × 10^−5^ M) induced a significant increase in dead cells (*p* < 0.001) with a slight increase in late apoptosis (*p* < 0.05) and decrease in living cells (*p* < 0.01; Fig. 4a). Moreover, the LDH/DNA ratio increased after 3 days of treatment with ketoconazole 5 × 10^−5^ M and 10^−5^ M (Fig. 4c left panel; *p* < 0.001; *p* < 0.05 respectively), while the apoptosis/DNA ratio increased only with the highest concentration tested (Fig. 4c, right panel; *p* < 0.001).

In BON-1 cells, the highest ketoconazole concentration tested (10^−5^ M) induced a significant increase in early apoptosis, as well as late apoptosis (*p* < 0.0001). Consequently, the percentage of live and dead cells decreased (Fig. 4b). In order to confirm these results, the percentage of LDH release was determined. After 3 days of treatment with ketoconazole 10^−5^ M, LDH increased by approximately 50%, combined with a threefold increase in apoptosis/DNA ratio (*p* < 0.05; Fig. 4d).

### Cell Cycle Progression

**Fig. 5:**
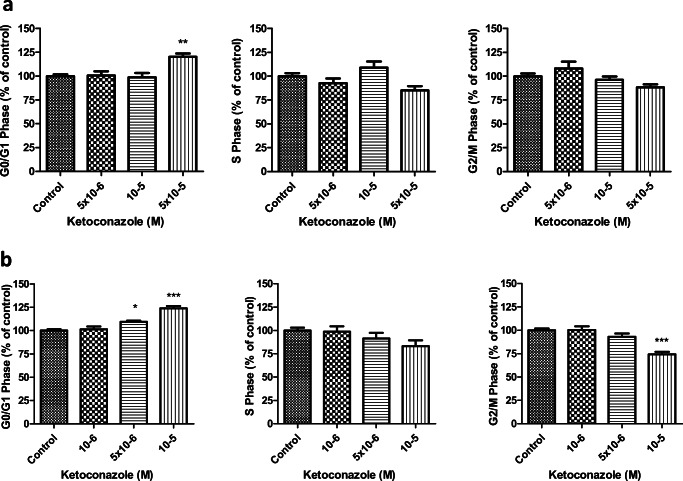
Dose-dependent effect of ketoconazole on cell cycle progression in DMS-79 cells (**a**) and BON-1 cells (**b**) after 3 days of treatment. In DMS-79 cells, ketoconazole (5 × 10^−5^ M) induced a slight but significant G1-phase increase. In BON-1 cells, ketoconazole (10^−5^–5 × 10^−6^ M) induced G1-phase increase and decrease in G2-phase. Asterisks *p < 0.05; **p < 0.01, ***p < 0.001 compared with untreated controls

In DMS-79, a slight statistically significant G0/G1-phase increase was observed with the highest ketoconazole concentration tested (*p* < 0.01), without having a statistical significant effect on the other cell cycle phases (Fig. 5a).

In BON-1 cell line, ketoconazole (10^−5^–5 × 10^−6^ M) induced a statistical significant G0/G1-phase increase (*p* < 0.0001, *p* < 0.01 respectively), accompanied by a decrease in G2-phase (*p* < 0.0001; Fig. 5b).

### Chromogranin A and POMC Expression

Ketoconazole did not have a statistically significant effect on POMC mRNA expression in DMS cells (Supplemental Figure 2A) nor on CgA mRNA expression in BON-1 cells (Supplemental Figure 2B) after 3 days of treatment at any of the doses tested.

### ACTH and Serotonin Secretion

**Fig. 6:**
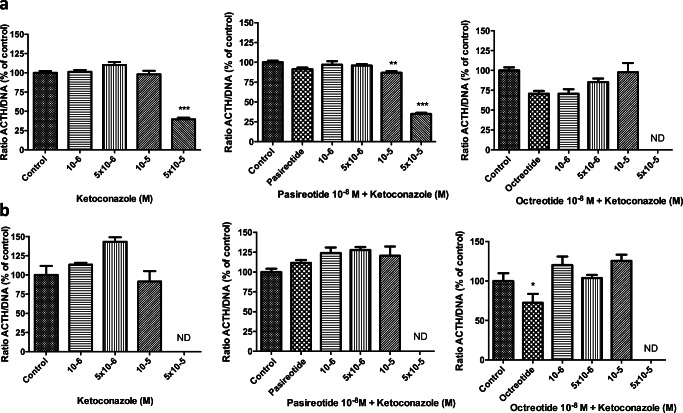
Dose-dependent effect of ketoconazole on ACTH secretion in DMS-79 cells after 3 (**a**) and 7 days (**b**) of treatment. Ketoconazole (5 × 10^−5^ M) significantly decreased ACTH secretion (corrected for cell number) after 3 days but not after 7 days of treatment, as well as its combination with pasireotide; octreotide decreased the ACTH/DNA ratio after 7 days. ACTH secretion was normalized to the effect on cell number (ratio ACTH/DNA). ND non-detectable ACTH. Asterisks *p < 0.05; **p < 0.01; ***p < 0.001, compared with untreated controls

**Fig. 7:**
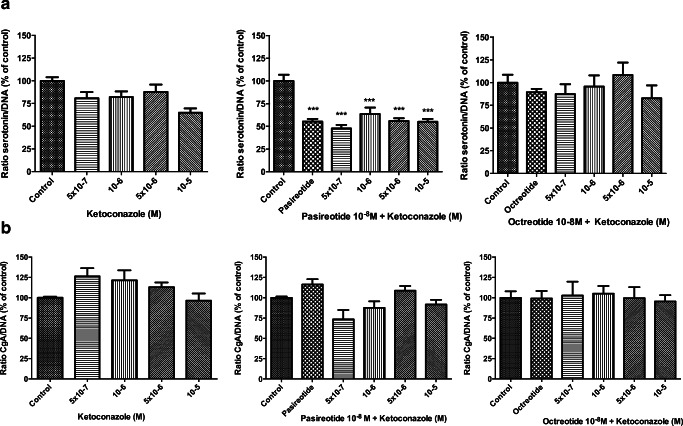
Dose-dependent effect of ketoconazole on serotonin (**a**) and chromogranin A (**b**) secretion in BON-1 cells after 3 days of treatment. In BON-1 cells, pasireotide reduced serotonin secretion; ketoconazole alone or in combination with octreotide did not significantly affected serotonin secretion. No statistically significant effect was observed on CgA secretion after treating cells with ketoconazole alone or in combination with pasireotide or octreotide. Serotonin and CgA secretion were normalized to the effect on cell number (ratio serotonin/DNA; ratio CgA/DNA). Asterisks ***p < 0.001, compared with untreated controls

Effects on ACTH secretion were evaluated in DMS-79 cells. When corrected for the effect of ketoconazole on total cell number (ACTH/DNA ratio), ketoconazole inhibited the release of ACTH after 3 days of treatment only with the highest tested concentration of ketoconazole (5 × 10–5 M) (Fig. 6a). ACTH was undetectable after 7 days of treatment with ketoconazole 5 × 10^−5^ M (Fig. 6b), likely due to the very potent lowering of cell number. When combined with pasireotide, a similar pattern of inhibition of ACTH release was observed (Fig. 6a and b, middle panels). Octreotide alone slightly inhibited ACTH release after 7 days of incubation. In combination with ketoconazole, only in the presence of the highest concentration of ketoconazole ACTH concentrations were undetectable (Fig. 6a and b, right panels). Supplementary Figure 3 shows the effect of ketoconazole on ACTH concentrations in the medium, not corrected for the effect of ketoconazole on cell number. It was not possible to measure serotonin secretion in BON-1 cells cultured in medium with FCS, due to a matrix effect in the serotonin assay (data not shown). In order to determine the effect of ketoconazole on serotonin secretion, BON-1 cells were cultured in medium containing 0.1% BSA. A dose-dependent inhibitory effect of ketoconazole on the proliferation of BON-1 cell line in 0.1% BSA medium was observed (Supplementary Figure 4A). The absolute production of serotonin decreased when cells were treated with high doses of ketoconazole, especially when combined with pasireotide, while no additional effect was observed when combined with octreotide (Supplementary Figure 4B). After calculating the serotonin/DNA ratio (to correct for the effect of ketoconazole on cell number), ketoconazole, and its combination with octreotide, did not have a statistical significant effect on serotonin secretion, while pasireotide decreased its production by 45% (Fig. 7a).

The absolute production of CgA in BON-1 cells decreased when cells were treated with high doses of ketoconazole (10^−5^–5 × 10^−6^ M). This effect remained when combined with pasireotide and octreotide (Supplementary Figure 4C). After calculating the CgA/DNA ratio, no statistically significant effect was observed after 3 days of treatment with ketoconazole alone or in combination with SSA (Fig. 7b).

## Discussion

In the present study, we evaluated the direct effects of ketoconazole in ACTH-producing and non-ACTH-producing NET cell lines. We examined effects on proliferation, mRNA expression, and secretion by ketoconazole as monotherapy, as well as in combination with SSA, the latter representing the recommended first-line therapy in non-functioning/functioning progressive G1/G2 NETs [31]. Our results revealed a particular effect of ketoconazole on proliferation, cell cycle, apoptosis, and mRNA expression, which was slightly different between the evaluated NET cell lines.

Ketoconazole is widely used for medical treatment of CS [10]. Apart from its inhibitory effect on steroidogenesis, ketoconazole may have direct antitumor effects as EAS cases have been described in which temporarily treatment with ketoconazole was followed by prolonged remission of hypercortisolemia [16]. For this reason, previously non-described off target in vitro effects of ketoconazole were evaluated in this study. While the evaluation of the effects of ketoconazole was the primary aim of this study, its combination with SSA was assessed due to their applicability in the clinical practice. SSAs are first-line therapy in functionally active NETs, including those associated with the carcinoid syndrome and functional pancreatic neuroendocrine tumors (PNETs) [32, 33]. We used two different SSA based on the fact that their anti-proliferative effect may depend on the expression level and type of SSTs in the tumor [34]. Since the affinity of pasireotide for SST_5_ is 30–40-fold higher when compared with octreotide [35], both SSA were selected for this study.

In our study, we included clinically relevant concentrations of ketoconazole, based on previous results in humans. Several studies report serum ketoconazole levels of 3.6 μg/mL and 6.5 μg/mL after the administration of 200 and 400 mg of ketoconazole respectively [36]. The continuous administration of 400 mg/8 h resulted in serum ketoconazole levels of 2–9 μg/mL [37]. Several effects in this study on the NET cell lines have been achieved with these ketoconazole concentrations (5.3 μg/mL ketoconazole corresponds to 10^−5^ M).

Sharma and Nieman described four patients with EAS with prolonged remission of hypercortisolism following treatment with ketoconazole alone or in combination with metyrapone and/or mitotane. In one patient, this was accompanied by increased ACTH levels indicating a direct, sustained suppressive effect on the adrenal cortex. In two other patients, however, ACTH levels were normal to low suggesting a direct effect of ketoconazole on ectopic ACTH-producing tumor cells [16]. In this sense, we observed decreased cell growth in both ACTH-producing and non-ACTH-producing NET cell lines, when exposed to ketoconazole in pharmacological concentrations (10^−5^–5 × 10^−6^ M). Importantly, DMS-79 cells are slightly less sensitive than BON-1 cells, and required higher doses for obtaining similar inhibitory effects in monolayer cultures. In the colony forming assay ketoconazole at a dose between 10^−5^ and 5 × 10^−6^ M induced similar cytotoxic effects in both cell lines.

The mechanism of action of ketoconazole seems to be different in both cell lines. In BON-1 cells, ketoconazole has an apoptotic effect (especially increasing early apoptosis), whereas in DMS-79 cell line, the effect seems to be more cytotoxic.

Previous reports have described a cytotoxic effect of ketoconazole in prostate cancer, adrenal cancer, and male metastatic breast cancer cell lines with a dose- and time-dependent pattern at clinically feasible concentrations [38]. In addition, it has been shown that ketoconazole decreased tumor surface area and tumor colonies in liver metastasis of human pancreatic adenocarcinoma in animal models [39]. This antitumor action of ketoconazole is further confirmed in our study, in which ketoconazole inhibited not only the number of colonies, but also the colony size. Due to its androgen lowering properties, ketoconazole is also evaluated as a potential treatment in other neoplasms such as prostate cancer [37]. In these patients, the drug is administered at high doses (400 mg/8 h/day), in combination with hydrocortisone [40]. Approximately, 27–60% of treated patients show up to 50% decrease in prostate antigen without receiving concomitant anti-androgen treatment. In addition, those patients that respond to treatment and do not have metastatic disease showed prolonged responses even after 7 years [40, 41]. Related to this, preliminary reports have suggested that the use of ketoconazole (600 mg/day) combined with docetaxel has a significant antitumor effect in castration-resistant prostate cancer patients [42]. The cell growth inhibitory effects observed in our study suggest that an off target direct antitumor action of ketoconazole may be involved in the sustained remission which is sometimes observed in patients with ectopic ACTH secreting NET that are treated with this drug [16].

Differences observed in sensitivity to ketoconazole between both cell lines may be related to the heterogeneity of NETs [2]. Tumor heterogeneity may explain different clinical responses to therapeutic options, including SSA and peptide receptor radionuclide therapy, as well as molecular targeted therapies [43–45]. In this sense, tumor heterogeneity affects clinical management and decision-making in NETs [46]. Additionally, tumor response may differ according to the primary tumor localization and tumor load [47]. DMS-79 and BON-1 are lung- and pancreas-derived cell lines, respectively. Furthermore, tumor cell characteristics were different; BON-1 cells attach to the culture plate surface, while DMS-79 grow in suspension and form small aggregates. Moreover, we observed a different somatostatin receptor expression pattern between both cell lines. Similar findings may be observed in other functional pathways that could also explain differences in the response of both cell lines [48]. Importantly, in the colony formation assay, DMS-79 cells were similarly sensitive to the effects of ketoconazole compared with BON-1 cells.

Focusing on treatment of NETs, it is already known that SSA have antitumor effects in patients with NET and that they are useful for symptoms control and disease stabilization [31, 49, 50]. Despite this, in vitro findings reveal a weak effect of SSA on cell proliferation [48]. In our study, we observed only a very weak anti-proliferative effect of octreotide after 3 days and of pasireotide after 7 days of treatment, which is concordant with reports in literature [31]. The response to pasireotide after 7 days in BON-1 cells is probably related to the high expression of SST_1_ and SST_5_ receptors in this cell line. Interestingly, in BON-1 cells, the effectiveness of ketoconazole in combination with SSA on cell proliferation seems to be slightly decreased.

Our results on apoptosis are concordant with previous studies in other cell line models. Ho et al. reported apoptosis induced by ketoconazole in human colorectal and hepatocellular carcinoma cell lines through the p53 pathway (increased *bax* and decreased *bcl-2* gene products) with minimal ketoconazole concentrations of 5 μg/mL [51]. Similarly, Won et al. suggested that ketoconazole could increase apoptosis and decrease cell viability in rats through reactive oxygen species (ROS) in rat cardiomyocytes [52]. Some other signaling routes have been proposed for explaining the apoptotic effect of ketoconazole, including the JNK phosphorylation in human osteosarcoma [53]. Ketoconazole, when combined with terfenadine, seems to potentiate the inhibition of cytochrome p450 3A4 in human cancer cell lines [54].

When evaluating cell cycle, published results are contradictory. Forgue-Lafitte et al. reported decreased cell number in S phase and a corresponding increase in G0-G1 phases in colon cancer cells treated with ketoconazole [55]. In contrast, Chen et al. described G0/G1 arrest in human colorectal and hepatocellular cell lines [56]. However, in our study, we observed in both cell lines a significant increase in G0/G1 phase, which was accompanied with an arrest of G2/M phases in BON-1 cells. These results suggest that ketoconazole may have a differential mechanism of action in different tumor cell lines.

When analyzing secretion, ketoconazole did not affect serotonin secretion, but the effect of pasireotide on serotonin production is relevant and stable after its combination with ketoconazole, suggesting a therapeutic indication for pasireotide in (a subset of) patients with carcinoid syndrome or functioning NETs with liver metastasis, refractory to classical SSA. Interestingly, pasireotide had no effect on CgA secretion, suggesting that serotonin may be a better marker of secretion. Pasireotide has a higher binding affinity for SST_1_, SST_3_, and SST_5_ when compared with octreotide, as well as a slightly lower affinity for SST_2_ [57]. Moreover, as we did not observe any effect of ketoconazole alone or combined with SSA on CgA secretion after correcting for its effect on cell number, it is likely that CgA is related to tumor growth and less to tumor secretion capacity [58].

Changes on hormone secretion by ketoconazole have been previously described. Ketoconazole increases the spontaneous and stimulated release of prolactin in normal and tumoral rat pituitary cells [59], and decreases the ACTH production in human thymic carcinoid cells [17]. It has been also hypothesized that ketoconazole could affect tumoral ACTH and cyclic ACTH secretion in vivo [16]. In our study, however, we did not observe a direct inhibitory effect of clinically feasible concentrations of ketoconazole on ACTH production by DMS-79 cells (corrected for the effect of ketoconazole on cell growth), suggesting that the effect of ketoconazole is primarily cytotoxic. The absence of an effect on ACTH production may also be reflected by the fact that ketoconazole did not affect POMC mRNA expression.

In summary, this study provides a primary comprehensive mapping of the effect of ketoconazole on NET cell lines, suggesting a potential effect of ketoconazole on cell proliferation, apoptosis, and cell cycle. This antitumor effect appears not tumor cell type–specific, since it was observed in both ACTH secreting and non-ACTH secreting NET cells. A direct antitumor action of ketoconazole may be an explanation for prolonged remission of hypercortisolemia observed in some patients with EAS and may have an added off target therapeutic value next to its adrenal-suppressive effects. Additional studies, including primary (ACTH-producing) NET cultures, are required to confirm and further extend these results.

## Electronic Supplementary Material

ESM 1 (PPTX 705 kb)
