# Supplementary figures and images for: Effects of Ketoconazole on ACTH-Producing and Non-ACTH-Producing Neuroendocrine Tumor Cells
Source: Horm Cancer. 2019 May 18;10(2-3):107–19. doi: 10.1007/s12672-019-00361-6 (PMC10355714; doi:10.1007/s12672-019-00361-6)

## Slide 1
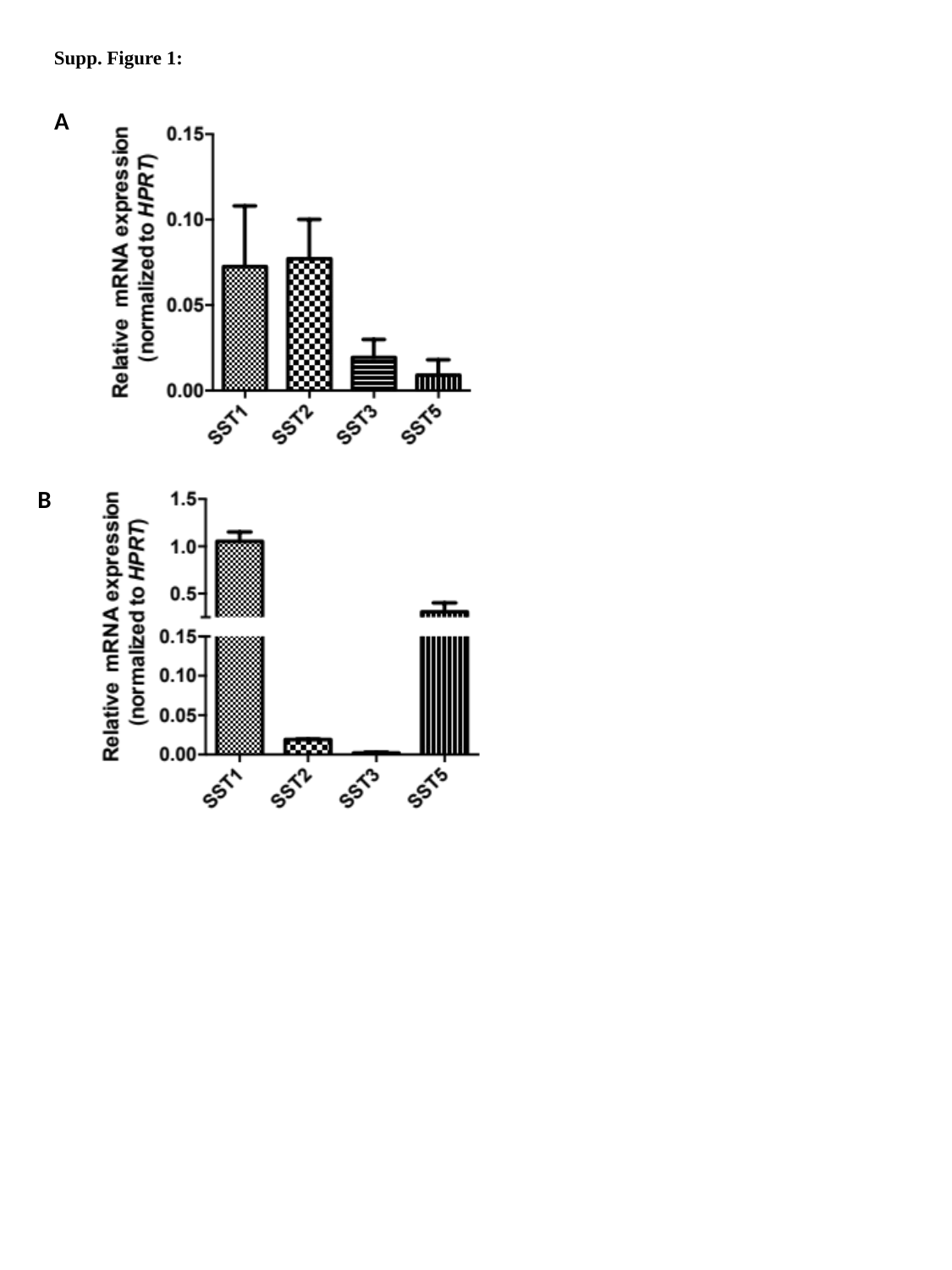

## Slide 2
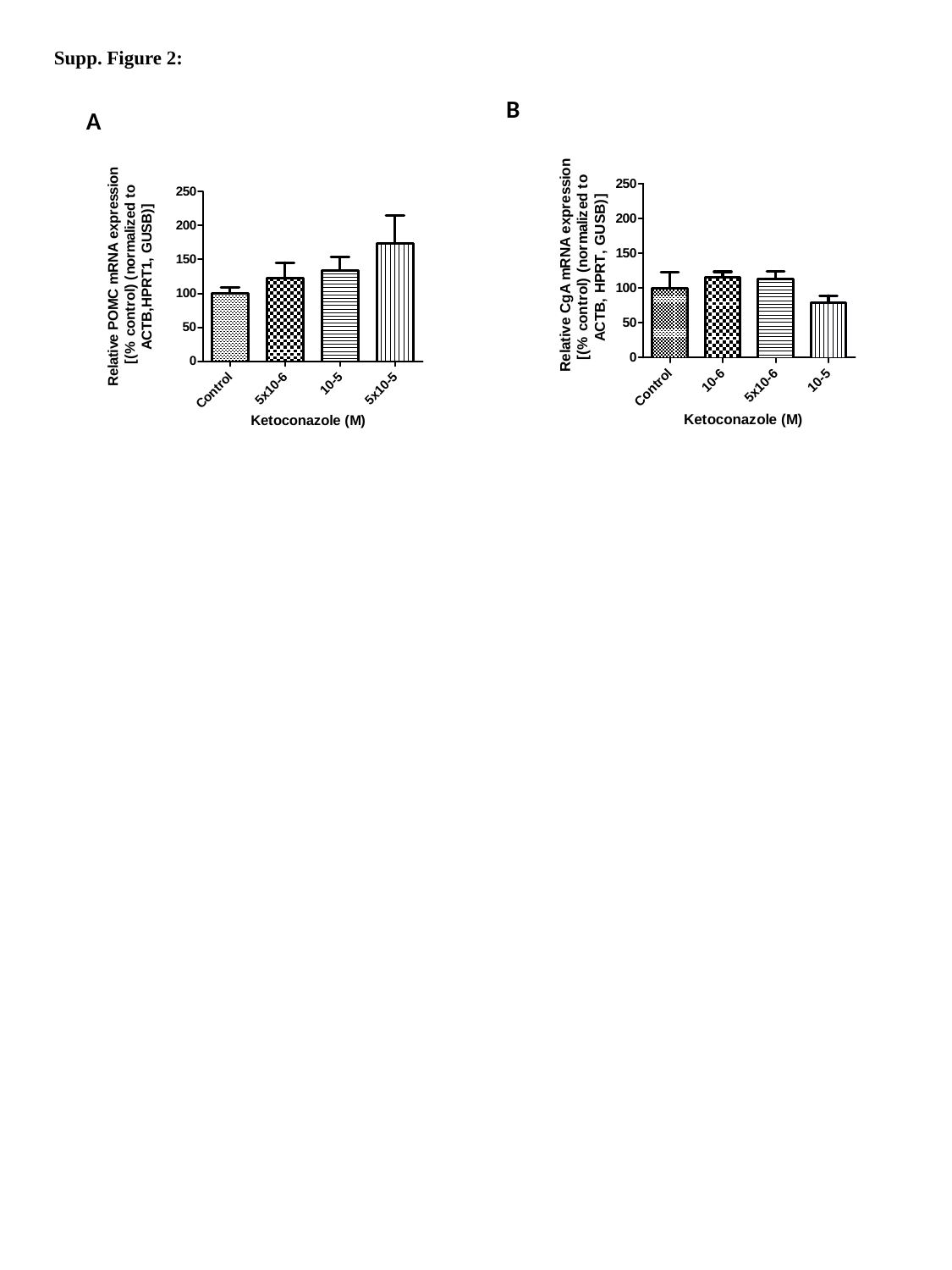

## Slide 3
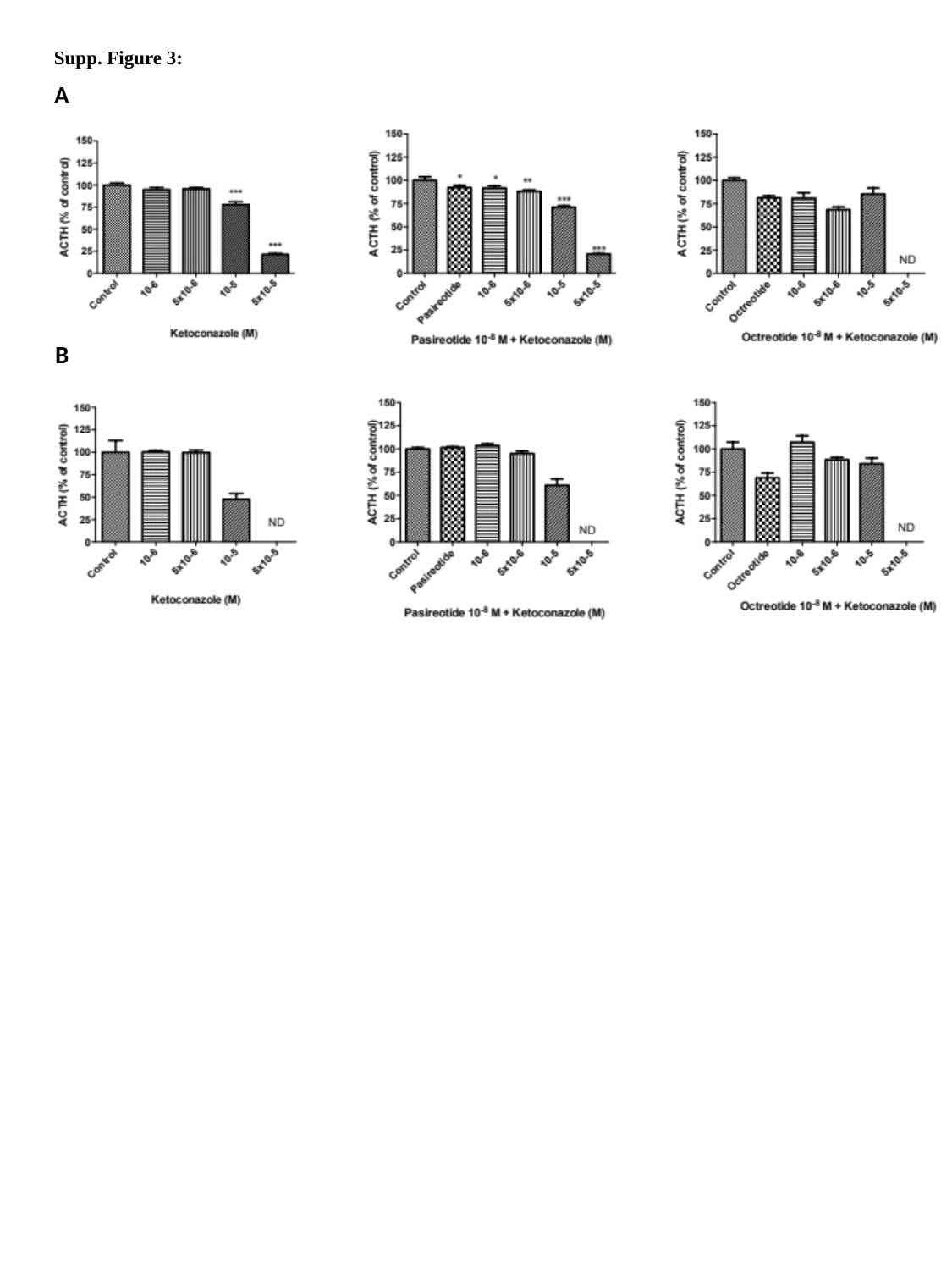

## Slide 4
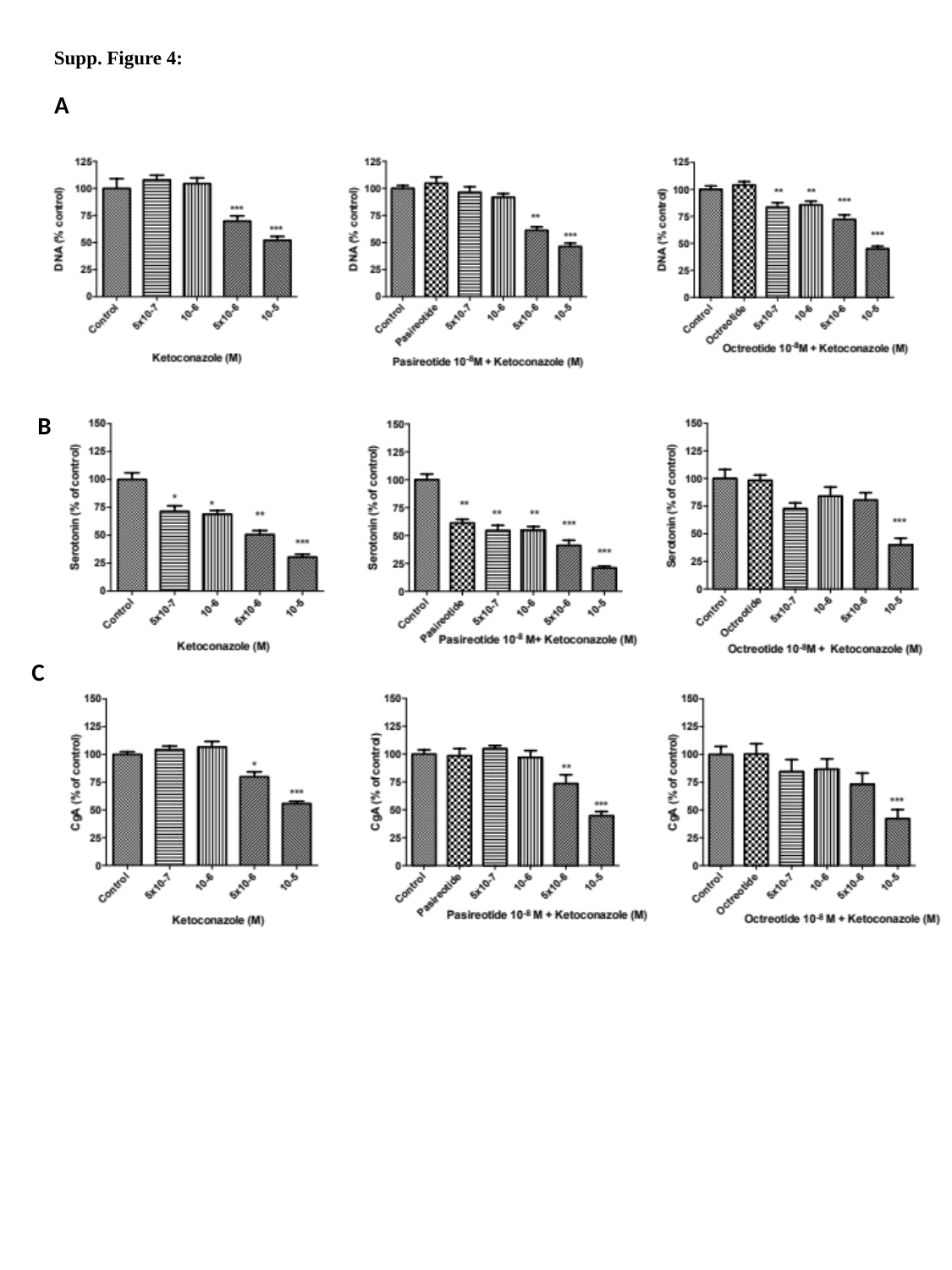

Supplement: Supplementary file 1 — (PPTX 705 kb) [file 12672_2019_361_MOESM1_ESM.pptx]
